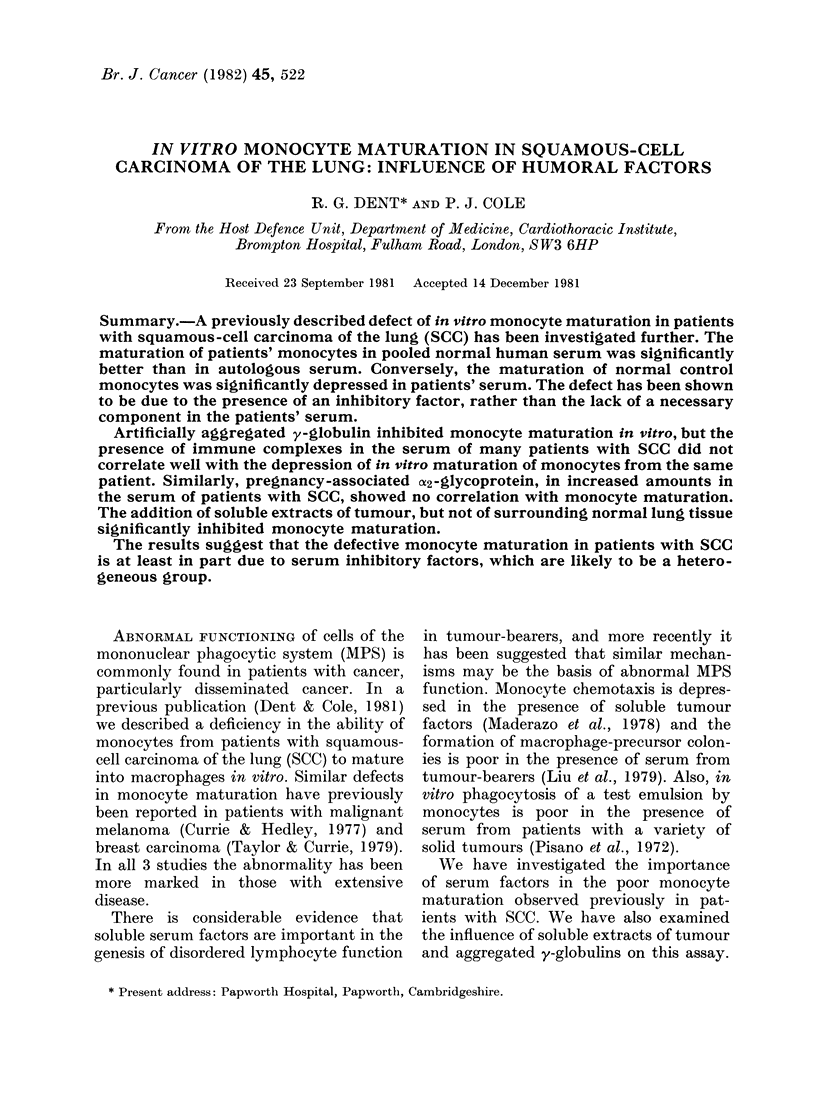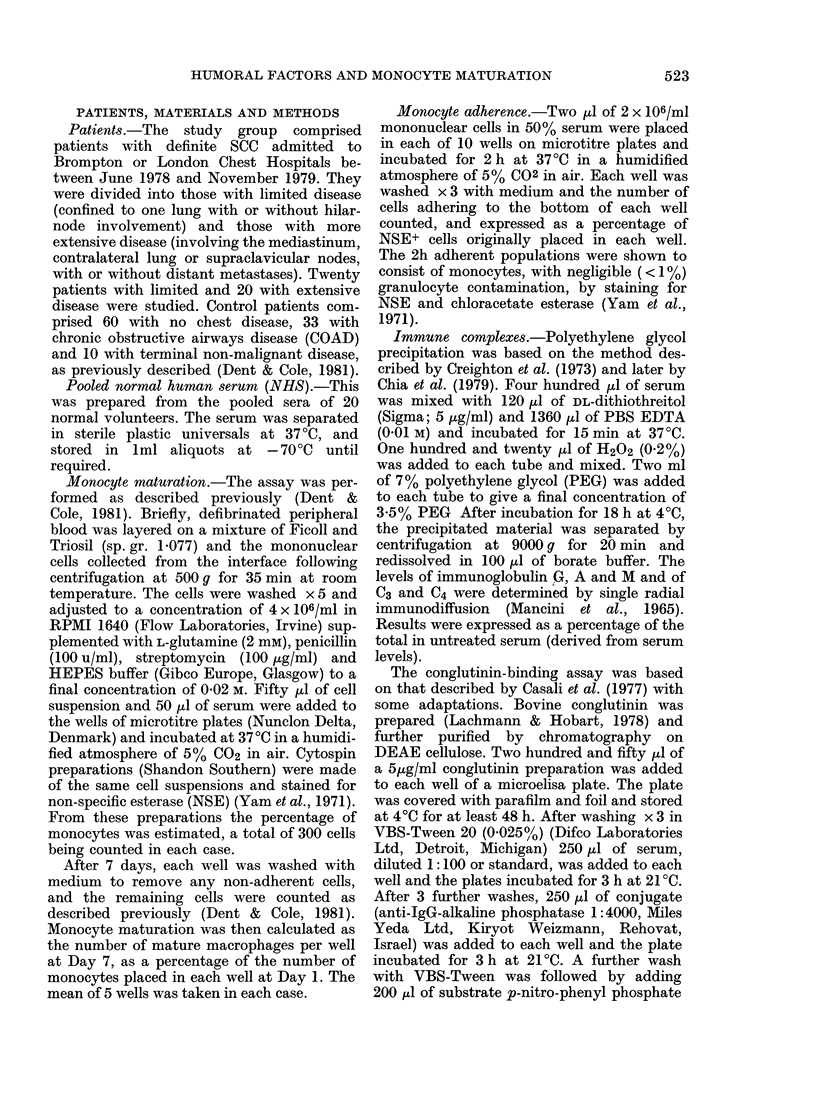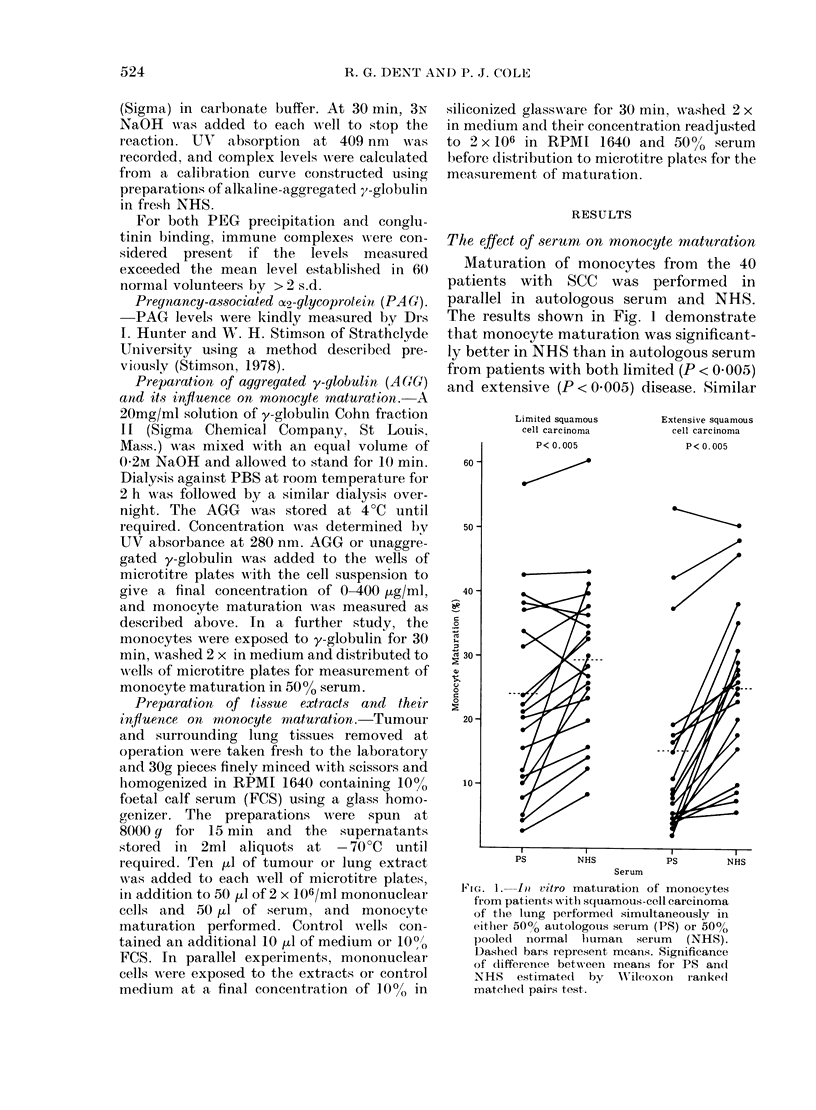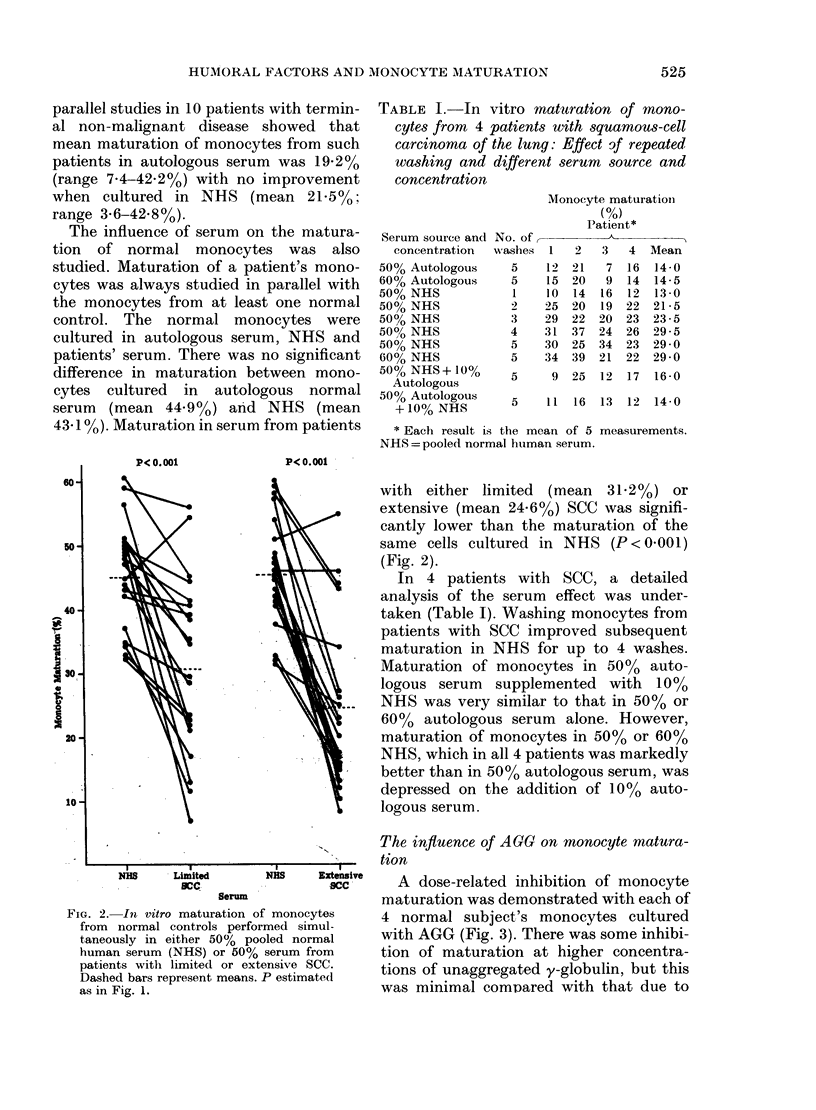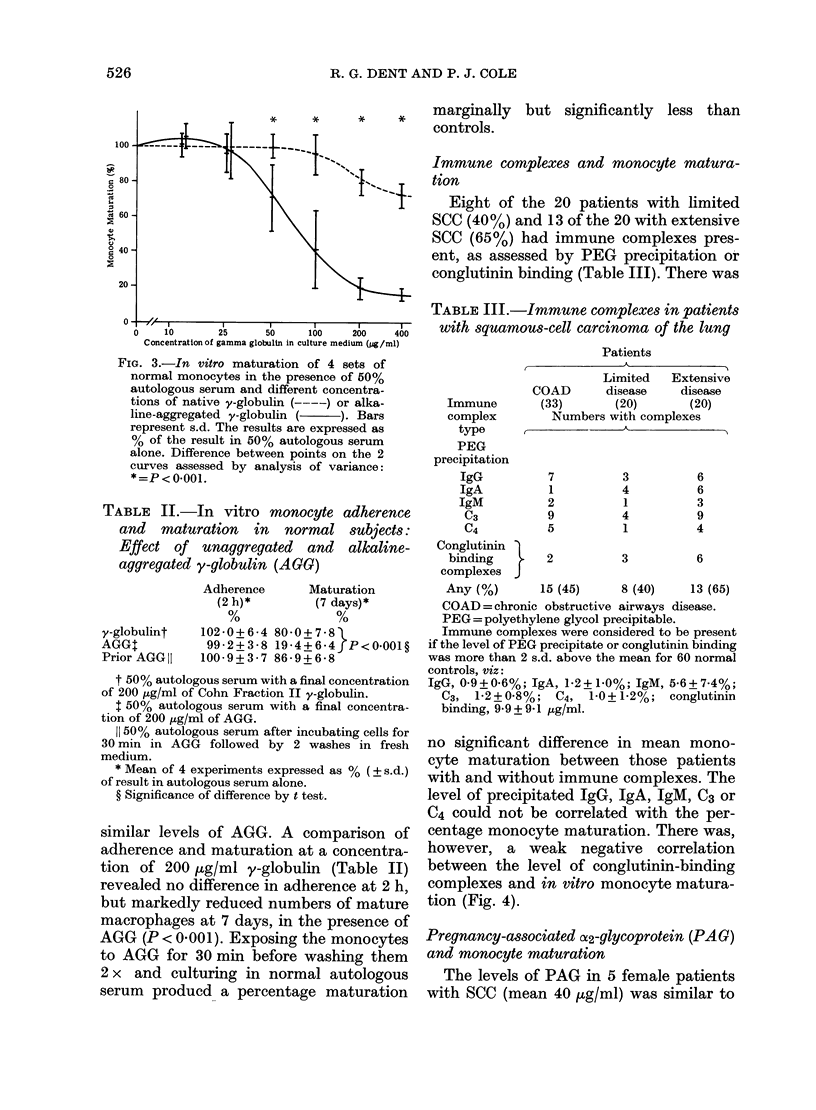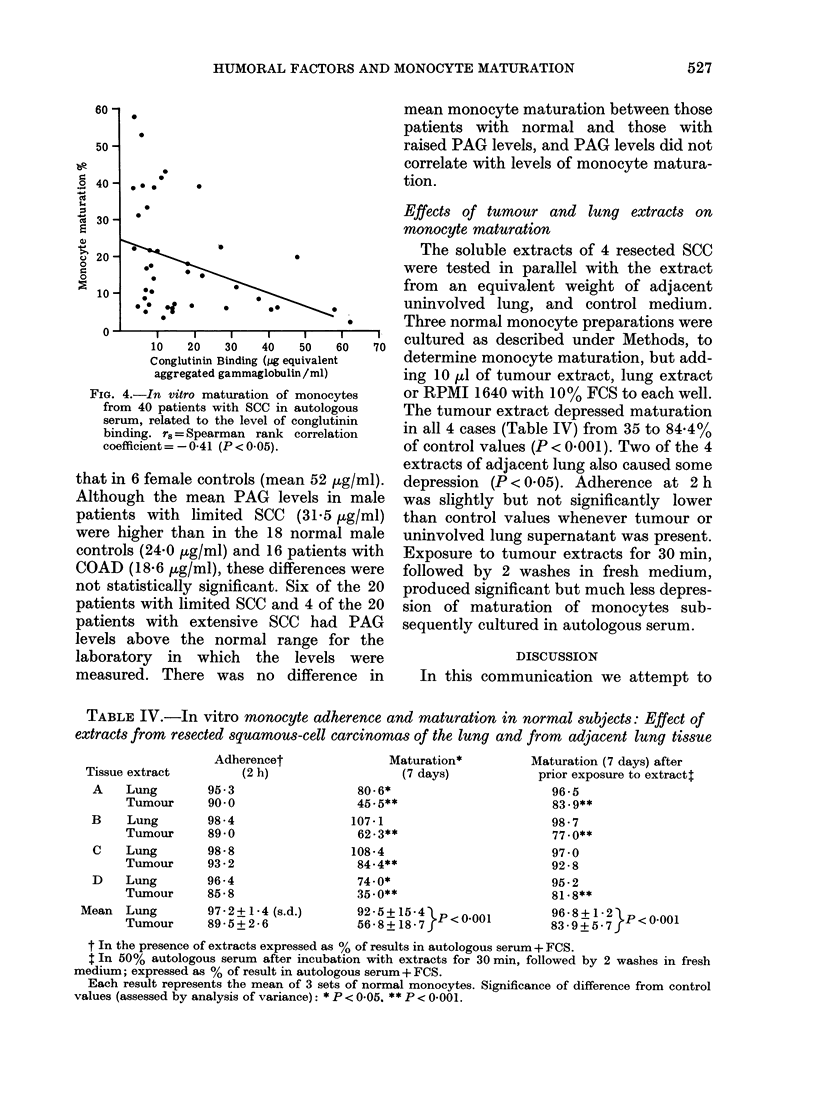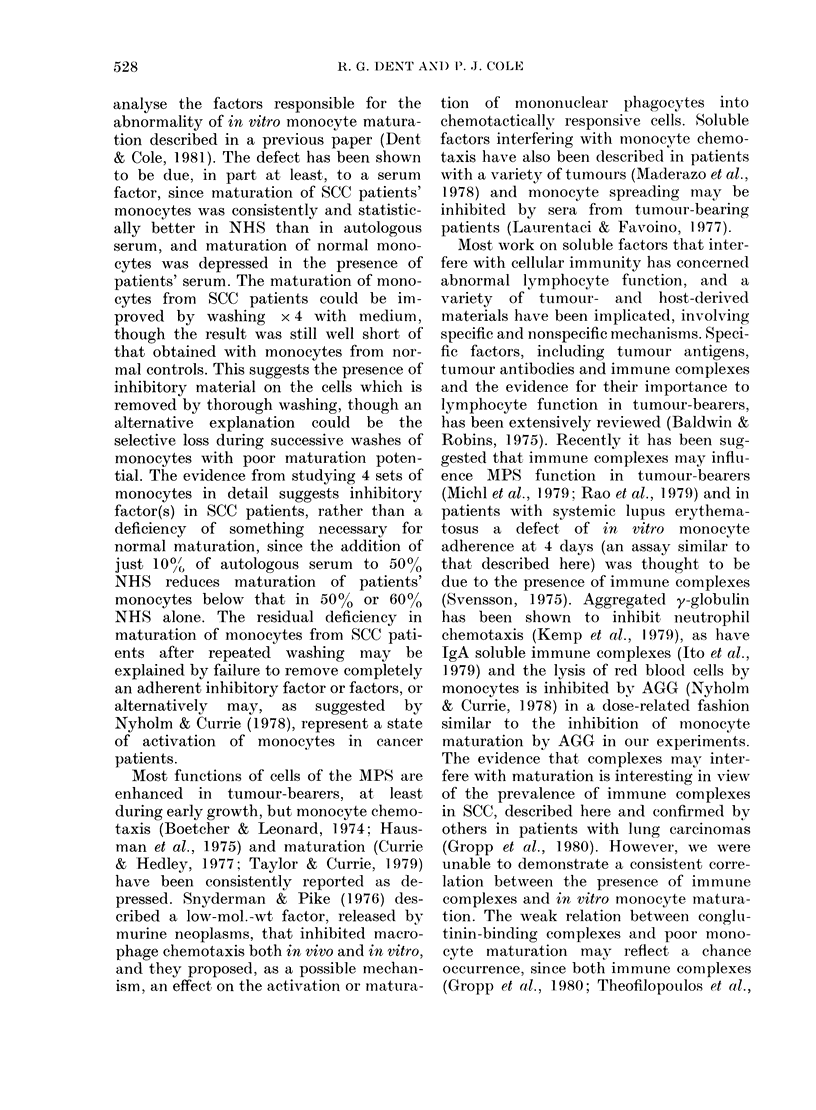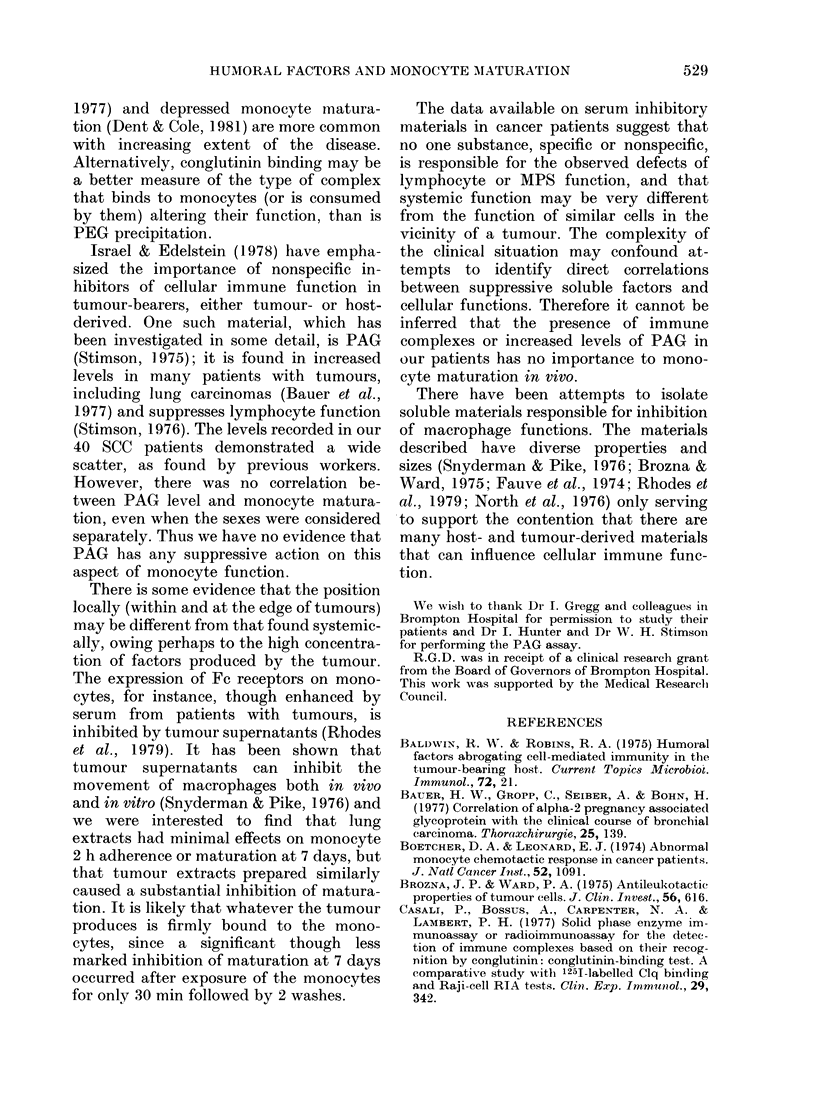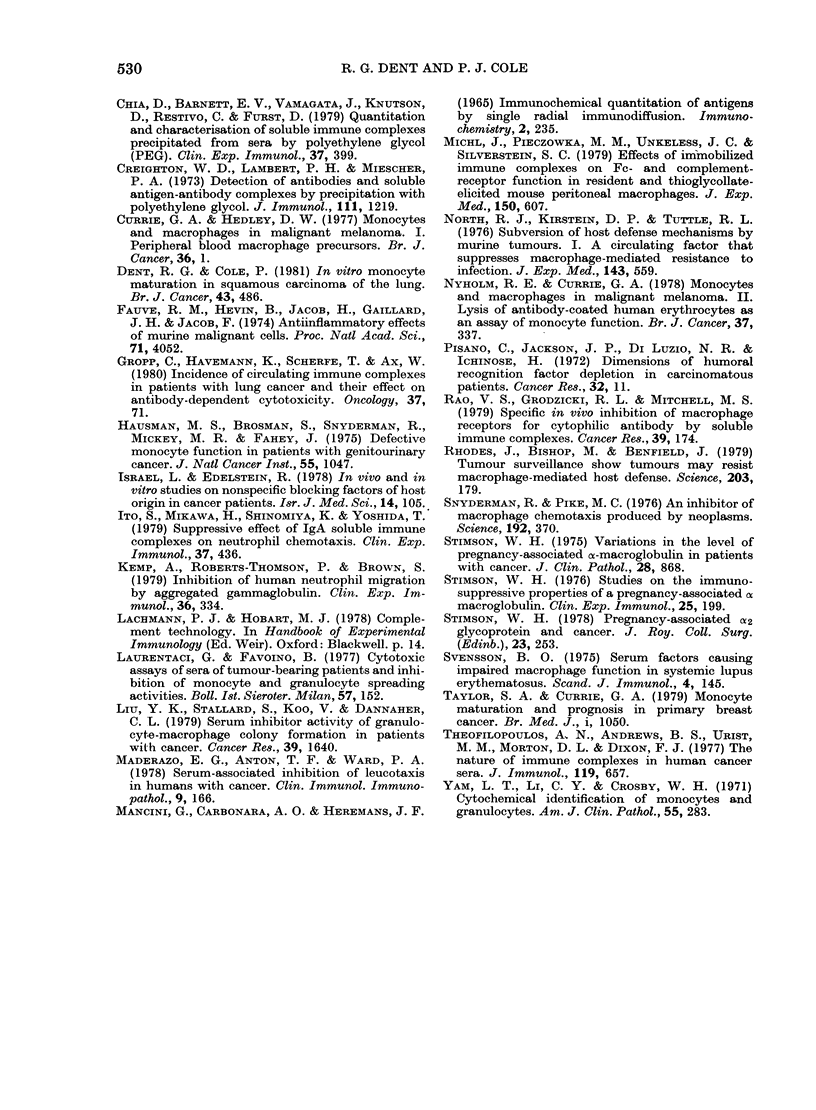# In vitro monocyte maturation in squamous-cell carcinoma of the lung: influence of humoral factors.

**DOI:** 10.1038/bjc.1982.88

**Published:** 1982-04

**Authors:** R. G. Dent, P. J. Cole

## Abstract

A previously described defect of in vitro monocyte maturation in patients with squamous-cell carcinoma of the lung (SCC) has been investigated further. The maturation of patients' monocytes in pooled normal human serum was significantly better than in autologous serum. Conversely, the maturation of normal control monocytes was significantly depressed in patients' serum. The defect has been shown to be due to the presence of an inhibitory factor, rather than the lack of a necessary component in the patients' serum. Artificially aggregated gamma-globulin inhibited monocyte maturation in vitro, but the presence of immune complexes in the serum of many patients with SCC did not correlate well with the depression of in vitro maturation of monocytes from the same patient. Similarly, pregnancy-associated alpha 2-glycoprotein, in increased amounts in the serum of patients with SCC, showed no correlation with monocyte maturation. The addition of soluble extracts of tumour, but not of surrounding normal lung tissue significantly inhibited monocyte maturation. The results suggest that the defective monocyte maturation in patients with SCC is at least in part due to serum inhibitory factors, which are likely to be a heterogeneous group.


*Br. J. Cancer* (1982) **45**, 522

### *IN VITRO* MONOCYTE MATURATION IN SQUAMOUS-CELL CARCINOMA OF THE LUNG: INFLUENCE OF HUMORAL FACTORS

R. G. DENT* AND P. J. COLE

*From the Host Defence Unit, Department of Medicine, Cardiothoracic Institute, Brompton Hospital, Fulham Road, London, SW3 6HP*

Received 23 September 1981 Accepted 14 December 1981

**Summary.—A previously described defect of *in vitro* monocyte maturation in patients with squamous-cell carcinoma of the lung (SCC) has been investigated further. The maturation of patients' monocytes in pooled normal human serum was significantly better than in autologous serum. Conversely, the maturation of normal control monocytes was significantly depressed in patients' serum. The defect has been shown to be due to the presence of an inhibitory factor, rather than the lack of a necessary component in the patients' serum.**

**Artificially aggregated $\gamma$-globulin inhibited monocyte maturation *in vitro*, but the presence of immune complexes in the serum of many patients with SCC did not correlate well with the depression of *in vitro* maturation of monocytes from the same patient. Similarly, pregnancy-associated $\alpha_2$-glycoprotein, in increased amounts in the serum of patients with SCC, showed no correlation with monocyte maturation. The addition of soluble extracts of tumour, but not of surrounding normal lung tissue significantly inhibited monocyte maturation.**

**The results suggest that the defective monocyte maturation in patients with SCC is at least in part due to serum inhibitory factors, which are likely to be a heterogeneous group.**

ABNORMAL FUNCTIONING of cells of the mononuclear phagocytic system (MPS) is commonly found in patients with cancer, particularly disseminated cancer. In a previous publication (Dent & Cole, 1981) we described a deficiency in the ability of monocytes from patients with squamous-cell carcinoma of the lung (SCC) to mature into macrophages *in vitro*. Similar defects in monocyte maturation have previously been reported in patients with malignant melanoma (Currie & Hedley, 1977) and breast carcinoma (Taylor & Currie, 1979). In all 3 studies the abnormality has been more marked in those with extensive disease.

There is considerable evidence that soluble serum factors are important in the genesis of disordered lymphocyte function in tumour-bearers, and more recently it has been suggested that similar mechanisms may be the basis of abnormal MPS function. Monocyte chemotaxis is depressed in the presence of soluble tumour factors (Maderazo *et al.*, 1978) and the formation of macrophage-precursor colonies is poor in the presence of serum from tumour-bearers (Liu *et al.*, 1979). Also, *in vitro* phagocytosis of a test emulsion by monocytes is poor in the presence of serum from patients with a variety of solid tumours (Pisano *et al.*, 1972).

We have investigated the importance of serum factors in the poor monocyte maturation observed previously in patients with SCC. We have also examined the influence of soluble extracts of tumour and aggregated $\gamma$-globulins on this assay.

* Present address: Papworth Hospital, Papworth, Cambridgeshire.

### PATIENTS, MATERIALS AND METHODS

*Patients*.—The study group comprised patients with definite SCC admitted to Brompton or London Chest Hospitals between June 1978 and November 1979. They were divided into those with limited disease (confined to one lung with or without hilar-node involvement) and those with more extensive disease (involving the mediastinum, contralateral lung or supraclavicular nodes, with or without distant metastases). Twenty patients with limited and 20 with extensive disease were studied. Control patients comprised 60 with no chest disease, 33 with chronic obstructive airways disease (COAD) and 10 with terminal non-malignant disease, as previously described (Dent & Cole, 1981).

*Pooled normal human serum* (*NHS*).—This was prepared from the pooled sera of 20 normal volunteers. The serum was separated in sterile plastic universals at 37°C, and stored in 1ml aliquots at −70°C until required.

*Monocyte maturation*.—The assay was performed as described previously (Dent & Cole, 1981). Briefly, defibrinated peripheral blood was layered on a mixture of Ficoll and Triosil (sp. gr. 1·077) and the mononuclear cells collected from the interface following centrifugation at 500 *g* for 35 min at room temperature. The cells were washed ×5 and adjusted to a concentration of $4 \times 10^6$/ml in RPMI 1640 (Flow Laboratories, Irvine) supplemented with L-glutamine (2 mM), penicillin (100 u/ml), streptomycin (100 μg/ml) and HEPES buffer (Gibco Europe, Glasgow) to a final concentration of 0·02 M. Fifty μl of cell suspension and 50 μl of serum were added to the wells of microtitre plates (Nunclon Delta, Denmark) and incubated at 37°C in a humidified atmosphere of 5% $CO_2$ in air. Cytospin preparations (Shandon Southern) were made of the same cell suspensions and stained for non-specific esterase (NSE) (Yam *et al*., 1971). From these preparations the percentage of monocytes was estimated, a total of 300 cells being counted in each case.

After 7 days, each well was washed with medium to remove any non-adherent cells, and the remaining cells were counted as described previously (Dent & Cole, 1981). Monocyte maturation was then calculated as the number of mature macrophages per well at Day 7, as a percentage of the number of monocytes placed in each well at Day 1. The mean of 5 wells was taken in each case.

*Monocyte adherence*.—Two μl of $2 \times 10^6$/ml mononuclear cells in 50% serum were placed in each of 10 wells on microtitre plates and incubated for 2 h at 37°C in a humidified atmosphere of 5% $CO^2$ in air. Each well was washed ×3 with medium and the number of cells adhering to the bottom of each well counted, and expressed as a percentage of $NSE^+$ cells originally placed in each well. The 2h adherent populations were shown to consist of monocytes, with negligible (<1%) granulocyte contamination, by staining for NSE and chloracetate esterase (Yam *et al*., 1971).

*Immune complexes*.—Polyethylene glycol precipitation was based on the method described by Creighton *et al*. (1973) and later by Chia *et al*. (1979). Four hundred μl of serum was mixed with 120 μl of DL-dithiothreitol (Sigma; 5 μg/ml) and 1360 μl of PBS EDTA (0·01 M) and incubated for 15 min at 37°C. One hundred and twenty μl of $H_2O_2$ (0·2%) was added to each tube and mixed. Two ml of 7% polyethylene glycol (PEG) was added to each tube to give a final concentration of 3·5% PEG After incubation for 18 h at 4°C, the precipitated material was separated by centrifugation at 9000 *g* for 20 min and redissolved in 100 μl of borate buffer. The levels of immunoglobulin G, A and M and of $C_3$ and $C_4$ were determined by single radial immunodiffusion (Mancini *et al*., 1965). Results were expressed as a percentage of the total in untreated serum (derived from serum levels).

The conglutinin-binding assay was based on that described by Casali *et al*. (1977) with some adaptations. Bovine conglutinin was prepared (Lachmann & Hobart, 1978) and further purified by chromatography on DEAE cellulose. Two hundred and fifty μl of a 5μg/ml conglutinin preparation was added to each well of a microelisa plate. The plate was covered with parafilm and foil and stored at 4°C for at least 48 h. After washing ×3 in VBS-Tween 20 (0·025%) (Difco Laboratories Ltd, Detroit, Michigan) 250 μl of serum, diluted 1:100 or standard, was added to each well and the plates incubated for 3 h at 21°C. After 3 further washes, 250 μl of conjugate (anti-IgG-alkaline phosphatase 1:4000, Miles Yeda Ltd, Kiryot Weizmann, Rehovat, Israel) was added to each well and the plate incubated for 3 h at 21°C. A further wash with VBS-Tween was followed by adding 200 μl of substrate *p*-nitro-phenyl phosphate

(Sigma) in carbonate buffer. At 30 min, 3N NaOH was added to each well to stop the reaction. UV absorption at 409 nm was recorded, and complex levels were calculated from a calibration curve constructed using preparations of alkaline-aggregated $\gamma$-globulin in fresh NHS.

For both PEG precipitation and conglutinin binding, immune complexes were considered present if the levels measured exceeded the mean level established in 60 normal volunteers by $>2$ s.d.

*Pregnancy-associated $\alpha_2$-glycoprotein (PAG).*—PAG levels were kindly measured by Drs I. Hunter and W. H. Stimson of Strathclyde University using a method described previously (Stimson, 1978).

*Preparation of aggregated $\gamma$-globulin (AGG) and its influence on monocyte maturation.*—A 20mg/ml solution of $\gamma$-globulin Cohn fraction II (Sigma Chemical Company, St Louis, Mass.) was mixed with an equal volume of 0·2M NaOH and allowed to stand for 10 min. Dialysis against PBS at room temperature for 2 h was followed by a similar dialysis overnight. The AGG was stored at 4°C until required. Concentration was determined by UV absorbance at 280 nm. AGG or unaggregated $\gamma$-globulin was added to the wells of microtitre plates with the cell suspension to give a final concentration of 0–400 $\mu$g/ml, and monocyte maturation was measured as described above. In a further study, the monocytes were exposed to $\gamma$-globulin for 30 min, washed 2 × in medium and distributed to wells of microtitre plates for measurement of monocyte maturation in 50% serum.

*Preparation of tissue extracts and their influence on monocyte maturation.*—Tumour and surrounding lung tissues removed at operation were taken fresh to the laboratory and 30g pieces finely minced with scissors and homogenized in RPMI 1640 containing 10% foetal calf serum (FCS) using a glass homogenizer. The preparations were spun at 8000 *g* for 15 min and the supernatants stored in 2ml aliquots at −70°C until required. Ten $\mu$l of tumour or lung extract was added to each well of microtitre plates, in addition to 50 $\mu$l of $2\times10^6$/ml mononuclear cells and 50 $\mu$l of serum, and monocyte maturation performed. Control wells contained an additional 10 $\mu$l of medium or 10% FCS. In parallel experiments, mononuclear cells were exposed to the extracts or control medium at a final concentration of 10% in siliconized glassware for 30 min, washed 2 × in medium and their concentration readjusted to $2\times10^6$ in RPMI 1640 and 50% serum before distribution to microtitre plates for the measurement of maturation.

### RESULTS

### *The effect of serum on monocyte maturation*

Maturation of monocytes from the 40 patients with SCC was performed in parallel in autologous serum and NHS. The results shown in Fig. 1 demonstrate that monocyte maturation was significantly better in NHS than in autologous serum from patients with both limited ($P<0{\cdot}005$) and extensive ($P<0{\cdot}005$) disease. Similar

FIG. 1.—*In vitro* maturation of monocytes from patients with squamous-cell carcinoma of the lung performed simultaneously in either 50% autologous serum (PS) or 50% pooled normal human serum (NHS). Dashed bars represent means. Significance of difference between means for PS and NHS estimated by Wilcoxon ranked matched pairs test.

parallel studies in 10 patients with terminal non-malignant disease showed that mean maturation of monocytes from such patients in autologous serum was 19·2% (range 7·4–42·2%) with no improvement when cultured in NHS (mean 21·5%; range 3·6–42·8%).

The influence of serum on the maturation of normal monocytes was also studied. Maturation of a patient's monocytes was always studied in parallel with the monocytes from at least one normal control. The normal monocytes were cultured in autologous serum, NHS and patients' serum. There was no significant difference in maturation between monocytes cultured in autologous normal serum (mean 44·9%) and NHS (mean 43·1%). Maturation in serum from patients

FIG. 2.—*In vitro* maturation of monocytes from normal controls performed simultaneously in either 50% pooled normal human serum (NHS) or 50% serum from patients with limited or extensive SCC. Dashed bars represent means. *P* estimated as in Fig. 1.

TABLE I.—In vitro *maturation of monocytes from 4 patients with squamous-cell carcinoma of the lung: Effect of repeated washing and different serum source and concentration*

| Serum source and concentration | No. of washes | Monocyte maturation (%) Patient* 1 | 2 | 3 | 4 | Mean |
|---|---|---|---|---|---|---|
| 50% Autologous | 5 | 12 | 21 | 7 | 16 | 14·0 |
| 60% Autologous | 5 | 15 | 20 | 9 | 14 | 14·5 |
| 50% NHS | 1 | 10 | 14 | 16 | 12 | 13·0 |
| 50% NHS | 2 | 25 | 20 | 19 | 22 | 21·5 |
| 50% NHS | 3 | 29 | 22 | 20 | 23 | 23·5 |
| 50% NHS | 4 | 31 | 37 | 24 | 26 | 29·5 |
| 50% NHS | 5 | 30 | 25 | 34 | 23 | 29·0 |
| 60% NHS | 5 | 34 | 39 | 21 | 22 | 29·0 |
| 50% NHS + 10% Autologous | 5 | 9 | 25 | 12 | 17 | 16·0 |
| 50% Autologous + 10% NHS | 5 | 11 | 16 | 13 | 12 | 14·0 |

* Each result is the mean of 5 measurements. NHS = pooled normal human serum.

with either limited (mean 31·2%) or extensive (mean 24·6%) SCC was significantly lower than the maturation of the same cells cultured in NHS ($P < 0{\cdot}001$) (Fig. 2).

In 4 patients with SCC, a detailed analysis of the serum effect was undertaken (Table I). Washing monocytes from patients with SCC improved subsequent maturation in NHS for up to 4 washes. Maturation of monocytes in 50% autologous serum supplemented with 10% NHS was very similar to that in 50% or 60% autologous serum alone. However, maturation of monocytes in 50% or 60% NHS, which in all 4 patients was markedly better than in 50% autologous serum, was depressed on the addition of 10% autologous serum.

### *The influence of AGG on monocyte maturation*

A dose-related inhibition of monocyte maturation was demonstrated with each of 4 normal subject's monocytes cultured with AGG (Fig. 3). There was some inhibition of maturation at higher concentrations of unaggregated $\gamma$-globulin, but this was minimal compared with that due to

FIG. 3.—*In vitro* maturation of 4 sets of normal monocytes in the presence of 50% autologous serum and different concentrations of native γ-globulin (----) or alkaline-aggregated γ-globulin (——). Bars represent s.d. The results are expressed as % of the result in 50% autologous serum alone. Difference between points on the 2 curves assessed by analysis of variance: $* = P < 0{\cdot}001$.

TABLE II.—In vitro *monocyte adherence and maturation in normal subjects: Effect of unaggregated and alkaline-aggregated γ-globulin (AGG)*

| | Adherence (2 h)* % | Maturation (7 days)* % | |
|---|---|---|---|
| γ-globulin† | 102·0±6·4 | 80·0±7·8 | $P < 0{\cdot}001$§ |
| AGG‡ | 99·2±3·8 | 19·4±6·4 | |
| Prior AGG‖ | 100·9±3·7 | 86·9±6·8 | |

† 50% autologous serum with a final concentration of 200 μg/ml of Cohn Fraction II γ-globulin.

‡ 50% autologous serum with a final concentration of 200 μg/ml of AGG.

‖ 50% autologous serum after incubating cells for 30 min in AGG followed by 2 washes in fresh medium.

* Mean of 4 experiments expressed as % (±s.d.) of result in autologous serum alone.

§ Significance of difference by *t* test.

similar levels of AGG. A comparison of adherence and maturation at a concentration of 200 μg/ml γ-globulin (Table II) revealed no difference in adherence at 2 h, but markedly reduced numbers of mature macrophages at 7 days, in the presence of AGG ($P < 0{\cdot}001$). Exposing the monocytes to AGG for 30 min before washing them 2× and culturing in normal autologous serum produced a percentage maturation marginally but significantly less than controls.

### *Immune complexes and monocyte maturation*

Eight of the 20 patients with limited SCC (40%) and 13 of the 20 with extensive SCC (65%) had immune complexes present, as assessed by PEG precipitation or conglutinin binding (Table III). There was

TABLE III.—*Immune complexes in patients with squamous-cell carcinoma of the lung*

| | Patients | | |
|---|---|---|---|
| Immune complex type | COAD (33) | Limited disease (20) | Extensive disease (20) |
| | Numbers with complexes | | |
| PEG precipitation | | | |
| IgG | 7 | 3 | 6 |
| IgA | 1 | 4 | 6 |
| IgM | 2 | 1 | 3 |
| $C_3$ | 9 | 4 | 9 |
| $C_4$ | 5 | 1 | 4 |
| Conglutinin binding complexes | 2 | 3 | 6 |
| Any (%) | 15 (45) | 8 (40) | 13 (65) |

COAD = chronic obstructive airways disease.
PEG = polyethylene glycol precipitable.
Immune complexes were considered to be present if the level of PEG precipitate or conglutinin binding was more than 2 s.d. above the mean for 60 normal controls, *viz*:
IgG, 0·9±0·6%; IgA, 1·2±1·0%; IgM, 5·6±7·4%; $C_3$, 1·2±0·8%; $C_4$, 1·0±1·2%; conglutinin binding, 9·9±9·1 μg/ml.

no significant difference in mean monocyte maturation between those patients with and without immune complexes. The level of precipitated IgG, IgA, IgM, $C_3$ or $C_4$ could not be correlated with the percentage monocyte maturation. There was, however, a weak negative correlation between the level of conglutinin-binding complexes and *in vitro* monocyte maturation (Fig. 4).

### *Pregnancy-associated $\alpha_2$-glycoprotein (PAG) and monocyte maturation*

The levels of PAG in 5 female patients with SCC (mean 40 μg/ml) was similar to

FIG. 4.—*In vitro* maturation of monocytes from 40 patients with SCC in autologous serum, related to the level of conglutinin binding. $r_s$ = Spearman rank correlation coefficient = −0·41 ($P < 0·05$).

that in 6 female controls (mean 52 μg/ml). Although the mean PAG levels in male patients with limited SCC (31·5 μg/ml) were higher than in the 18 normal male controls (24·0 μg/ml) and 16 patients with COAD (18·6 μg/ml), these differences were not statistically significant. Six of the 20 patients with limited SCC and 4 of the 20 patients with extensive SCC had PAG levels above the normal range for the laboratory in which the levels were measured. There was no difference in mean monocyte maturation between those patients with normal and those with raised PAG levels, and PAG levels did not correlate with levels of monocyte maturation.

*Effects of tumour and lung extracts on monocyte maturation*

The soluble extracts of 4 resected SCC were tested in parallel with the extract from an equivalent weight of adjacent uninvolved lung, and control medium. Three normal monocyte preparations were cultured as described under Methods, to determine monocyte maturation, but adding 10 μl of tumour extract, lung extract or RPMI 1640 with 10% FCS to each well. The tumour extract depressed maturation in all 4 cases (Table IV) from 35 to 84·4% of control values ($P < 0·001$). Two of the 4 extracts of adjacent lung also caused some depression ($P < 0·05$). Adherence at 2 h was slightly but not significantly lower than control values whenever tumour or uninvolved lung supernatant was present. Exposure to tumour extracts for 30 min, followed by 2 washes in fresh medium, produced significant but much less depression of maturation of monocytes subsequently cultured in autologous serum.

### DISCUSSION

In this communication we attempt to

TABLE IV.—In vitro *monocyte adherence and maturation in normal subjects: Effect of extracts from resected squamous-cell carcinomas of the lung and from adjacent lung tissue*

| Tissue extract | | Adherence† (2 h) | Maturation* (7 days) | Maturation (7 days) after prior exposure to extract‡ |
|---|---|---|---|---|
| A | Lung | 95·3 | 80·6* | 96·5 |
| | Tumour | 90·0 | 45·5** | 83·9** |
| B | Lung | 98·4 | 107·1 | 98·7 |
| | Tumour | 89·0 | 62·3** | 77·0** |
| C | Lung | 98·8 | 108·4 | 97·0 |
| | Tumour | 93·2 | 84·4** | 92·8 |
| D | Lung | 96·4 | 74·0* | 95·2 |
| | Tumour | 85·8 | 35·0** | 81·8** |
| Mean | Lung | 97·2 ± 1·4 (s.d.) | 92·5 ± 15·4 } $P < 0·001$ | 96·8 ± 1·2 } $P < 0·001$ |
| | Tumour | 89·5 ± 2·6 | 56·8 ± 18·7 | 83·9 ± 5·7 |

† In the presence of extracts expressed as % of results in autologous serum + FCS.
‡ In 50% autologous serum after incubation with extracts for 30 min, followed by 2 washes in fresh medium; expressed as % of result in autologous serum + FCS.
Each result represents the mean of 3 sets of normal monocytes. Significance of difference from control values (assessed by analysis of variance): * $P < 0·05$. ** $P < 0·001$.

analyse the factors responsible for the abnormality of *in vitro* monocyte maturation described in a previous paper (Dent & Cole, 1981). The defect has been shown to be due, in part at least, to a serum factor, since maturation of SCC patients' monocytes was consistently and statistically better in NHS than in autologous serum, and maturation of normal monocytes was depressed in the presence of patients' serum. The maturation of monocytes from SCC patients could be improved by washing ×4 with medium, though the result was still well short of that obtained with monocytes from normal controls. This suggests the presence of inhibitory material on the cells which is removed by thorough washing, though an alternative explanation could be the selective loss during successive washes of monocytes with poor maturation potential. The evidence from studying 4 sets of monocytes in detail suggests inhibitory factor(s) in SCC patients, rather than a deficiency of something necessary for normal maturation, since the addition of just 10% of autologous serum to 50% NHS reduces maturation of patients' monocytes below that in 50% or 60% NHS alone. The residual deficiency in maturation of monocytes from SCC patients after repeated washing may be explained by failure to remove completely an adherent inhibitory factor or factors, or alternatively may, as suggested by Nyholm & Currie (1978), represent a state of activation of monocytes in cancer patients.

Most functions of cells of the MPS are enhanced in tumour-bearers, at least during early growth, but monocyte chemotaxis (Boetcher & Leonard, 1974; Hausman *et al.*, 1975) and maturation (Currie & Hedley, 1977; Taylor & Currie, 1979) have been consistently reported as depressed. Snyderman & Pike (1976) described a low-mol.-wt factor, released by murine neoplasms, that inhibited macrophage chemotaxis both *in vivo* and *in vitro*, and they proposed, as a possible mechanism, an effect on the activation or maturation of mononuclear phagocytes into chemotactically responsive cells. Soluble factors interfering with monocyte chemotaxis have also been described in patients with a variety of tumours (Maderazo *et al.*, 1978) and monocyte spreading may be inhibited by sera from tumour-bearing patients (Laurentaci & Favoino, 1977).

Most work on soluble factors that interfere with cellular immunity has concerned abnormal lymphocyte function, and a variety of tumour- and host-derived materials have been implicated, involving specific and nonspecific mechanisms. Specific factors, including tumour antigens, tumour antibodies and immune complexes and the evidence for their importance to lymphocyte function in tumour-bearers, has been extensively reviewed (Baldwin & Robins, 1975). Recently it has been suggested that immune complexes may influence MPS function in tumour-bearers (Michl *et al.*, 1979; Rao *et al.*, 1979) and in patients with systemic lupus erythematosus a defect of *in vitro* monocyte adherence at 4 days (an assay similar to that described here) was thought to be due to the presence of immune complexes (Svensson, 1975). Aggregated $\gamma$-globulin has been shown to inhibit neutrophil chemotaxis (Kemp *et al.*, 1979), as have IgA soluble immune complexes (Ito *et al.*, 1979) and the lysis of red blood cells by monocytes is inhibited by AGG (Nyholm & Currie, 1978) in a dose-related fashion similar to the inhibition of monocyte maturation by AGG in our experiments. The evidence that complexes may interfere with maturation is interesting in view of the prevalence of immune complexes in SCC, described here and confirmed by others in patients with lung carcinomas (Gropp *et al.*, 1980). However, we were unable to demonstrate a consistent correlation between the presence of immune complexes and *in vitro* monocyte maturation. The weak relation between conglutinin-binding complexes and poor monocyte maturation may reflect a chance occurrence, since both immune complexes (Gropp *et al.*, 1980; Theofilopoulos *et al.*,

1977) and depressed monocyte maturation (Dent & Cole, 1981) are more common with increasing extent of the disease. Alternatively, conglutinin binding may be a better measure of the type of complex that binds to monocytes (or is consumed by them) altering their function, than is PEG precipitation.

Israel & Edelstein (1978) have emphasized the importance of nonspecific inhibitors of cellular immune function in tumour-bearers, either tumour- or host-derived. One such material, which has been investigated in some detail, is PAG (Stimson, 1975); it is found in increased levels in many patients with tumours, including lung carcinomas (Bauer *et al.*, 1977) and suppresses lymphocyte function (Stimson, 1976). The levels recorded in our 40 SCC patients demonstrated a wide scatter, as found by previous workers. However, there was no correlation between PAG level and monocyte maturation, even when the sexes were considered separately. Thus we have no evidence that PAG has any suppressive action on this aspect of monocyte function.

There is some evidence that the position locally (within and at the edge of tumours) may be different from that found systemically, owing perhaps to the high concentration of factors produced by the tumour. The expression of Fc receptors on monocytes, for instance, though enhanced by serum from patients with tumours, is inhibited by tumour supernatants (Rhodes *et al.*, 1979). It has been shown that tumour supernatants can inhibit the movement of macrophages both *in vivo* and *in vitro* (Snyderman & Pike, 1976) and we were interested to find that lung extracts had minimal effects on monocyte 2 h adherence or maturation at 7 days, but that tumour extracts prepared similarly caused a substantial inhibition of maturation. It is likely that whatever the tumour produces is firmly bound to the monocytes, since a significant though less marked inhibition of maturation at 7 days occurred after exposure of the monocytes for only 30 min followed by 2 washes.

The data available on serum inhibitory materials in cancer patients suggest that no one substance, specific or nonspecific, is responsible for the observed defects of lymphocyte or MPS function, and that systemic function may be very different from the function of similar cells in the vicinity of a tumour. The complexity of the clinical situation may confound attempts to identify direct correlations between suppressive soluble factors and cellular functions. Therefore it cannot be inferred that the presence of immune complexes or increased levels of PAG in our patients has no importance to monocyte maturation *in vivo*.

There have been attempts to isolate soluble materials responsible for inhibition of macrophage functions. The materials described have diverse properties and sizes (Snyderman & Pike, 1976; Brozna & Ward, 1975; Fauve *et al.*, 1974; Rhodes *et al.*, 1979; North *et al.*, 1976) only serving to support the contention that there are many host- and tumour-derived materials that can influence cellular immune function.

We wish to thank Dr I. Gregg and colleagues in Brompton Hospital for permission to study their patients and Dr I. Hunter and Dr W. H. Stimson for performing the PAG assay.

R.G.D. was in receipt of a clinical research grant from the Board of Governors of Brompton Hospital. This work was supported by the Medical Research Council.

### REFERENCES

Baldwin, R. W. & Robins, R. A. (1975) Humoral factors abrogating cell-mediated immunity in the tumour-bearing host. *Current Topics Microbiol. Immunol.*, **72**, 21.

Bauer, H. W., Gropp, C., Seiber, A. & Bohn, H. (1977) Correlation of alpha-2 pregnancy associated glycoprotein with the clinical course of bronchial carcinoma. *Thoraxchirurgie*, **25**, 139.

Boetcher, D. A. & Leonard, E. J. (1974) Abnormal monocyte chemotactic response in cancer patients. *J. Natl Cancer Inst.*, **52**, 1091.

Brozna, J. P. & Ward, P. A. (1975) Antileukotactic properties of tumour cells. *J. Clin. Invest.*, **56**, 616.

Casali, P., Bossus, A., Carpenter, N. A. & Lambert, P. H. (1977) Solid phase enzyme immunoassay or radioimmunoassay for the detection of immune complexes based on their recognition by conglutinin: conglutinin-binding test. A comparative study with $^{125}$I-labelled Clq binding and Raji-cell RIA tests. *Clin. Exp. Immunol.*, **29**, 342.

CHIA, D., BARNETT, E. V., VAMAGATA, J., KNUTSON, D., RESTIVO, C. & FURST, D. (1979) Quantitation and characterisation of soluble immune complexes precipitated from sera by polyethylene glycol (PEG). *Clin. Exp. Immunol.*, **37**, 399.

CREIGHTON, W. D., LAMBERT, P. H. & MIESCHER, P. A. (1973) Detection of antibodies and soluble antigen-antibody complexes by precipitation with polyethylene glycol. *J. Immunol.*, **111**, 1219.

CURRIE, G. A. & HEDLEY, D. W. (1977) Monocytes and macrophages in malignant melanoma. I. Peripheral blood macrophage precursors. *Br. J. Cancer*, **36**, 1.

DENT, R. G. & COLE, P. (1981) *In vitro* monocyte maturation in squamous carcinoma of the lung. *Br. J. Cancer*, **43**, 486.

FAUVE, R. M., HEVIN, B., JACOB, H., GAILLARD, J. H. & JACOB, F. (1974) Antiinflammatory effects of murine malignant cells. *Proc. Natl Acad. Sci.*, **71**, 4052.

GROPP, C., HAVEMANN, K., SCHERFE, T. & AX, W. (1980) Incidence of circulating immune complexes in patients with lung cancer and their effect on antibody-dependent cytotoxicity. *Oncology*, **37**, 71.

HAUSMAN, M. S., BROSMAN, S., SNYDERMAN, R., MICKEY, M. R. & FAHEY, J. (1975) Defective monocyte function in patients with genitourinary cancer. *J. Natl Cancer Inst.*, **55**, 1047.

ISRAEL, L. & EDELSTEIN, R. (1978) *In vivo* and *in vitro* studies on nonspecific blocking factors of host origin in cancer patients. *Isr. J. Med. Sci.*, **14**, 105.

ITO, S., MIKAWA, H., SHINOMIYA, K. & YOSHIDA, T. (1979) Suppressive effect of IgA soluble immune complexes on neutrophil chemotaxis. *Clin. Exp. Immunol.*, **37**, 436.

KEMP, A., ROBERTS-THOMSON, P. & BROWN, S. (1979) Inhibition of human neutrophil migration by aggregated gammaglobulin. *Clin. Exp. Immunol.*, **36**, 334.

LACHMANN, P. J. & HOBART, M. J. (1978) Complement technology. In *Handbook of Experimental Immunology* (Ed. Weir). Oxford: Blackwell. p. 14.

LAURENTACI, G. & FAVOINO, B. (1977) Cytotoxic assays of sera of tumour-bearing patients and inhibition of monocyte and granulocyte spreading activities. *Boll. Ist. Sieroter. Milan*, **57**, 152.

LIU, Y. K., STALLARD, S., KOO, V. & DANNAHER, C. L. (1979) Serum inhibitor activity of granulocyte-macrophage colony formation in patients with cancer. *Cancer Res.*, **39**, 1640.

MADERAZO, E. G., ANTON, T. F. & WARD, P. A. (1978) Serum-associated inhibition of leucotaxis in humans with cancer. *Clin. Immunol. Immunopathol.*, **9**, 166.

MANCINI, G., CARBONARA, A. O. & HEREMANS, J. F. (1965) Immunochemical quantitation of antigens by single radial immunodiffusion. *Immunochemistry*, **2**, 235.

MICHL, J., PIECZOWKA, M. M., UNKELESS, J. C. & SILVERSTEIN, S. C. (1979) Effects of immobilized immune complexes on Fc- and complement-receptor function in resident and thioglycollate-elicited mouse peritoneal macrophages. *J. Exp. Med.*, **150**, 607.

NORTH, R. J., KIRSTEIN, D. P. & TUTTLE, R. L. (1976) Subversion of host defense mechanisms by murine tumours. I. A circulating factor that suppresses macrophage-mediated resistance to infection. *J. Exp. Med.*, **143**, 559.

NYHOLM, R. E. & CURRIE, G. A. (1978) Monocytes and macrophages in malignant melanoma. II. Lysis of antibody-coated human erythrocytes as an assay of monocyte function. *Br. J. Cancer*, **37**, 337.

PISANO, C., JACKSON, J. P., DI LUZIO, N. R. & ICHINOSE, H. (1972) Dimensions of humoral recognition factor depletion in carcinomatous patients. *Cancer Res.*, **32**, 11.

RAO, V. S., GRODZICKI, R. L. & MITCHELL, M. S. (1979) Specific *in vivo* inhibition of macrophage receptors for cytophilic antibody by soluble immune complexes. *Cancer Res.*, **39**, 174.

RHODES, J., BISHOP, M. & BENFIELD, J. (1979) Tumour surveillance show tumours may resist macrophage-mediated host defense. *Science*, **203**, 179.

SNYDERMAN, R. & PIKE, M. C. (1976) An inhibitor of macrophage chemotaxis produced by neoplasms. *Science*, **192**, 370.

STIMSON, W. H. (1975) Variations in the level of pregnancy-associated $\alpha$-macroglobulin in patients with cancer. *J. Clin. Pathol.*, **28**, 868.

STIMSON, W. H. (1976) Studies on the immunosuppressive properties of a pregnancy-associated $\alpha$ macroglobulin. *Clin. Exp. Immunol.*, **25**, 199.

STIMSON, W. H. (1978) Pregnancy-associated $\alpha_2$ glycoprotein and cancer. *J. Roy. Coll. Surg. (Edinb.)*, **23**, 253.

SVENSSON, B. O. (1975) Serum factors causing impaired macrophage function in systemic lupus erythematosus. *Scand. J. Immunol.*, **4**, 145.

TAYLOR, S. A. & CURRIE, G. A. (1979) Monocyte maturation and prognosis in primary breast cancer. *Br. Med. J.*, i, 1050.

THEOFILOPOULOS, A. N., ANDREWS, B. S., URIST, M. M., MORTON, D. L. & DIXON, F. J. (1977) The nature of immune complexes in human cancer sera. *J. Immunol.*, **119**, 657.

YAM, L. T., LI, C. Y. & CROSBY, W. H. (1971) Cytochemical identification of monocytes and granulocytes. *Am. J. Clin. Pathol.*, **55**, 283.